# Understanding decision making for the use of atraumatic restorative approach based on non-clinical factors by Indian pedodontists - a conjoint analysis.

**DOI:** 10.12688/f1000research.157561.1

**Published:** 2024-11-21

**Authors:** Bhuvaneshwari Nadar, Usha GV, Sultan Almalki, Inderjit Gowdar

**Affiliations:** 1 Public Health Dentistry, TPCT's Terna Dental College, Nerul, Navi Mumbai, Maharashtra, 400706, India; 2 Public Health Dentistry, Bapuji Dental College and Hospital, Davangere, KARNATAKA, 577004, India; 3 Preventive Dental Sciences, College of Dentistry Prince Sattam bin Abdul Aziz University, Al-kharj, KSA, Saudi Arabia

**Keywords:** atraumatic restorative treatment, conjoint analysis, dillmans technique, response rate, decision making

## Abstract

**Background:**

Atraumatic Restorative Treatment (ART) approach is considered as one of the minimally invasive interventions. The success of restoration depends on various clinical factors like material, patient and operator factor. Conjoint analysis is a technique for measuring individual’s preference structures via systematical variations of products attributes in an experimental design.

**Aim:**

Assess Pedodontists’ perception of non-clinical factors and investigate influence of child’s age, level of cooperation and vulnerability status on their decisions to perform Atraumatic Restorative Approach (ART) approach using hypothetical patient scenario’s and conjoint design.

**Materials and methods:**

A cross-sectional exploratory survey using a web-based questionnaire was performed among Pedodontists registered as life members under Indian Association of Pedodontic and Preventive Dentistry. Self-designed structured scenario-based questionnaire was prepared and validated. Three non-clinical factors Age of child, Cooperation level and Vulnerability status were considered. Using orthogonal design, SPSS software nine profiles were created randomly along with two holdouts. Final study proforma consisted of three sections with description of eleven clinical scenarios. It was administered to participants using Google forms. Using SPSS conjoint software relative utilities for each factor on decision for using ART was estimated.

**Results:**

Four hundred and thirty-two pedodontists had completed the survey (35.9%). Pedodontists considered vulnerability status of the child as the most important factor for their decision to use ART treatment. For the vulnerability status, the factor vulnerable had the greatest utility (-0.364) compared to non-vulnerable factor (0.364). For cooperation level, factor uncooperative had greater utility (-0.343), as compared to moderately cooperative (0.066) and uncooperative (0.277). For the age factor, the age of 4 years had the greatest utility (-0.175) compared with age 6 (-0.013) and age 12 (0.19).

**Conclusion:**

The most preferred scenario by pedodontists to consider ART as treatment of choice was child belonging to vulnerable section, being very young and uncooperative.

## Introduction

Minimum intervention dentistry can be defined as a philosophy of professional care concerned with the first occurrence, earliest detection and possible cure of diseases on micro levels, followed by minimally invasive and patient friendly treatment to repair irreversible damage caused by such disease. ^1^ Atraumatic Restorative Treatment (ART) is a key technique in this approach, has gained significant interest recently, especially in addressing dental care access challenges. ^2^ Originally developed for use in developing countries with limited resources, ART has proven effective in treating primary dentition, achieving high success rates. ^3^ Its adoption in developed countries has primarily focused on managing severe early childhood caries. ART utilizes fluoride-releasing glass ionomer materials, which help control caries progression while providing a patient-friendly, minimally invasive approach. This method not only facilitates care in resource-limited settings but also supports preventive strategies in more affluent areas, highlighting its versatility and effectiveness in managing progressive dental caries. ^4^

A meta-analysis conducted in 2021 evaluated the success rates of Atraumatic Restorative Treatment (ART) in both primary and permanent dentitions. The findings indicated impressive success rates at the 12-month follow-up mark: 71% for ART restorations on primary teeth and 96% for those on permanent teeth, without influenced by setting. These results underscore ART’s effectiveness as a reliable treatment option, reinforcing its role in minimally invasive dentistry, especially for managing caries in diverse patient populations. ^5^

The success of dental restorations, including Atraumatic Restorative treatment (ART) is influenced by a variety of clinical factors. Among these, the operator’s decisions are crucial. Key operator factors include the diagnostic methods used, the dentist’s preferences and beliefs about restoration longevity and effectiveness, as well as their skills and experience. ^6^ The choice of materials and the nature of the intervention also play significant roles. For example, a dentist’s restorative decisions may depend on the patient’s age, behavioral compliance, caries risk, and socioeconomic background. ^7^ These factors shape how the dentist approaches treatment, ultimately impacting the overall success of the restoration. Therefore, a comprehensive understanding of both clinical and patient-specific variables is essential for optimizing treatment outcomes in dentistry. ^8^

One of the most common methods for assessing dental restorative treatment decisions is through survey research. In these surveys, respondents answer direct questions about their preferences and the importance of various factors that influencing their choices of dental procedures. However, this method typically evaluates individual factors in isolation and does not capture the complexity of “derived decisions,” which consider multiple factors simultaneously. ^9^ This limitation can affect the validity of the data and insights derived from such surveys, making it essential to consider alternative methods or complementary approaches to gain a more comprehensive understanding of decision-making in dental restorative treatments. ^10^

In the early 1970s, Green and Rao introduced conjoint measurement to marketing research to better understand and predict buyer behavior. ^11^ The term “conjoint” reflects the concept that multiple factors can be evaluated together, ^12^ allowing individuals to make choices among different hypothetical products or treatment scenarios rather than assessing each characteristic in isolation. Recently, the popularity of conjoint analysis has grown in health outcomes research. ^13^ Conjoint analysis has seen limited application in dental research, but its potential is being recognized. ^9, 10, 14, 15^ One notable study utilized conjoint analysis to evaluate dentists’ decisions regarding implant treatment based on various patient characteristics. ^10^ This research revealed differences between dentists’ stated preferences and their derived decisions, suggesting that conjoint analysis can more accurately reflect actual decision-making processes by requiring respondents to make trade-offs in a choice context.

Despite its advantages, studies examining dentists’ use of specific techniques and the factors influencing their decisions remain relatively scarce. As the field continues to evolve, applying conjoint analysis could provide deeper insights into how various attributes impact treatment decisions, ultimately leading to more informed and patient-centered care in dentistry.

Dentist’s clinical decision making regarding restorative interventions for the treatment of caries vary significantly. ^16^ The ART approach, has been recognized as an economical and effective method for managing carious lesions, in vulnerable population. ^17^ Decisions to use ART often depend on social and environmental factors, including a child’s age, socioeconomic status, and level of cooperation. ^18^

Despite ART’s relevance, there has been no research assessing the perceptions of Indian pedodontists. This study aims to explore how these non-clinical factors influence pedodontists’ willingness to adopt the ART approach, using hypothetical patient scenarios and a conjoint design. The central research question investigates whether differences exist in clinical decision-making among pedodontists regarding ART while considering these non-clinical factors?

## Methods

The study was designed, analyzed, and interpreted according to the Conjoint Analysis applications in the health checklist. ^19^

### Participants

A cross-sectional exploratory survey was conducted among pedodontists using a web-based questionnaire. All the Pedodontists registered as life members under the Indian Association of Pedodontic and Preventive Dentistry and who were willing to participate were included.

### Instrument

The first stage in the design of a conjoint study is to choose attributes and assign levels.

1.
*Defining attributes*

  An attribute is a characteristic of a product (e.g., color), made up of various levels (there must be at least two for each attribute) or degrees of that characteristic (e.g., red, yellow, blue). ^20^ Conjoint analysis elicits preferences for a decision over the range of attributes and levels that define the hypothetical scenarios was used in questions. The three non-clinical factors or attributes selected for the present study were patient age, level of cooperation, and vulnerability status. These were selected based on a literature review and expert opinions. These attributes were refined through consultations with experts from the Department of Pedodontics and Public Health Dentistry. The modified attributes chosen were found to be meaningful to test and relevant to understand their interplay for clinical decisions by dentists.
2.
*Assigning levels*

  For the chosen attributes, levels were assigned, providing a realistic profile/scenario capable of being traded. ^14^ Attribute levels were identified after a literature review and discussion with experts. For age four, six and 12 years, which coincide with the three important stages, that is, primary, mixed dentition, and 12 years, the global indicator age group was selected. For the child cooperation level, Frankl’s behavior rating scale, the first and second categories (definitively negative and negative) were clubbed together and considered as uncooperative; the third category positive was modified as moderately cooperative and the fourth category definitively positive was modified as cooperative for convenience. The third factor, vulnerability status, was classified as vulnerable or non-vulnerable. Children who attended public schools, orphans, or were either physically or mentally challenged were considered vulnerable. Attending private schools, physically and mentally healthy, and living with parents as non-vulnerable.

Considering the three non-clinical factors, the possible permutation combination of the scenarios/profiles was 18. According to literature, all the Profiles should be included in the study, but by doing so it can cause fatigue and boredom set in. ^14^ Therefore, by using Fractional Factorial Design the number of paired comparisons were reduced to the smallest number necessary for efficient estimation of utility weights. ^21^ Using orthogonal design in SPSS software, half fraction (nine Profiles) were created randomly. The software algorithm was programmed to extrapolate the total utility values for all possible patient scenarios for each study participant, although a subset of scenarios was used.

The self-designed structured web-based questionnaire had three sections: a) nine conjoint questions, with the definition of ART, and a detailed explanation regarding the clinical presentation of the carious lesion in all scenarios. Clinical conditions were fixed for all scenarios with carious lesions involving the primary or permanent molar, with the depth of the lesion within dentin and no pulpal involvement clinically or radiographically. A description regarding the three non-clinical factors that varied in all scenarios are described. Pedodontists were requested to rate how likely they were to use ART on a 5 – point likert scale, ranging from 5 (very unlikely to use ART) to 1 (very likely to use ART). b) Two questions focused on whether pedodontists consider ART to be definitive or interim treatment and why, as well as a question asking where they learned about ART. c) The last three questions on demographic and practice characteristics. The questionnaire was pretested for content validity by two faculty members, each from the Department of Public Health Dentistry and the Department of Pedodontics. The CVI score is 0.88, suggesting good content validity. ^22^

### Administration of the questionnaire and data collection

A web-based survey questionnaire with the help of Google Forms was administered to all pedodontists registered as life members. The questionnaire was in the form of a booklet with multiple sections containing the covering letter, its usefulness and importance of the respondent response, online consent form, the survey questions, and the thank you note with participants’ suggestions regarding the study and their opinions. An invitation email was sent to all participants to 2-3 days prior to the actual email of the survey. Personalized emails that included a link to the web survey. Three reminder mails were sent after the first, fourth, and seventh week to improve the response rate. The remaining mails included a covering letter and a replacement questionnaire, emphasizing the respondent’s importance and explanation of the identification number and how confidentiality is protected and a thank you message. ^23^

### Analysis

SPSS conjoint software [IBM Corp. in Armonk, NY] ( https://www.ibm.com/spss) was used to estimate the relative weights (utilities) for each level of the presented factors in the decision to use ART. Linear regression was used to generate the utility scores for each attribute level.

## Results

**Figure 1.**
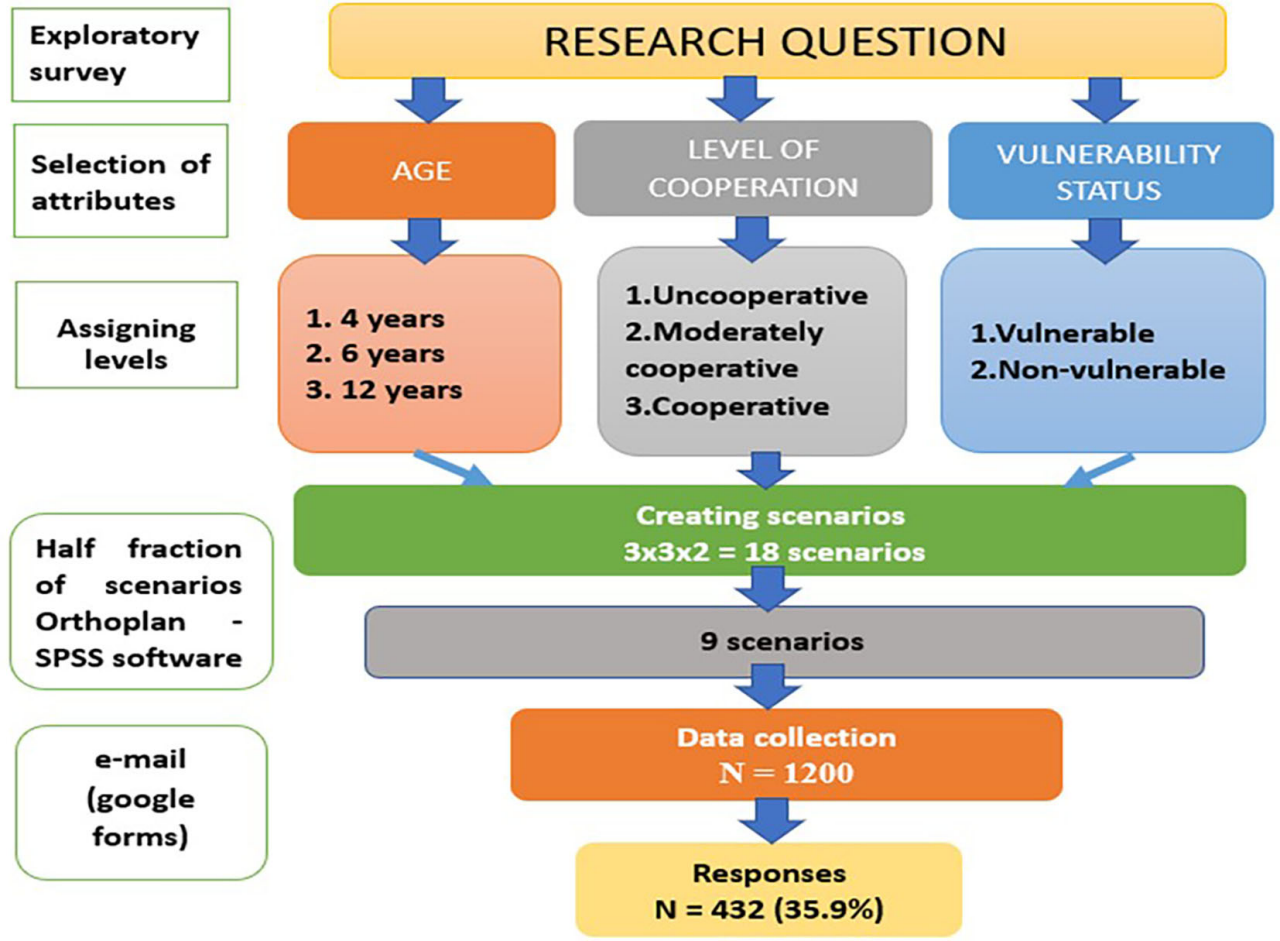
Flowchart of the study.

**Figure 2.**
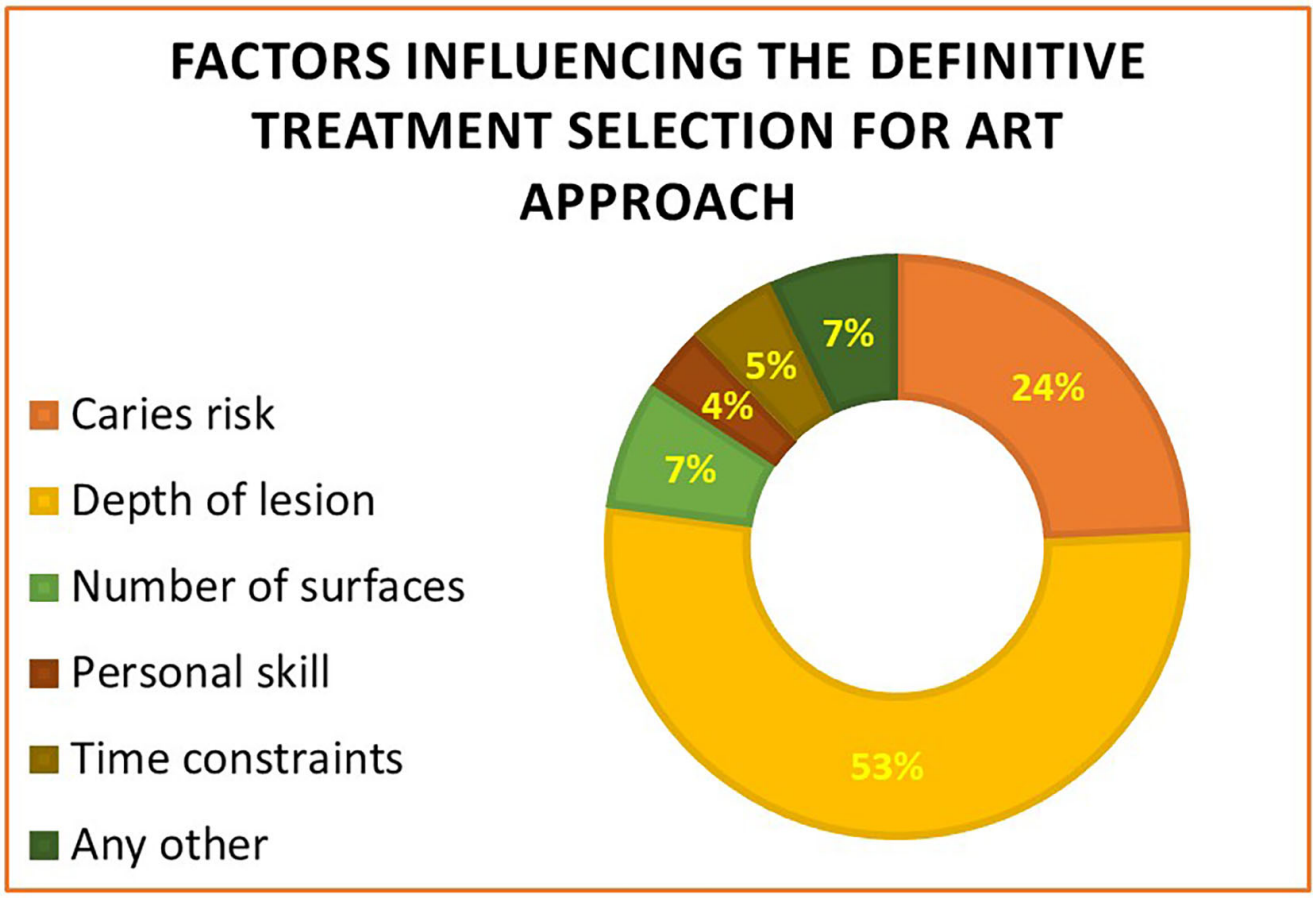
Factors influencing the definitive treatment selection for art approach.

In the present survey, 432 pedodontists have responded, with a response rate of 35.9% ( Figure 1). They were predominantly female 62%. About 32% were into academics and private practitioners, 28% private practitioners, 30% postgraduate students, and 10% were only academics. More than half of the respondents (62%) reported ART as interim treatment and 38% as definitive for the given scenarios. Factors included depth of lesion (53%), caries risk (24%), personal skill (4%), time constraints (5%), number of surfaces, and other reasons (7%) (see Figure 2). Pedodontists reported, they had learned ART during undergraduate courses (45.3%), postgraduation (24.7%), textbooks and articles (23.3%), and continuing dental education programs and conferences (6.7%).

**Figure 3.**
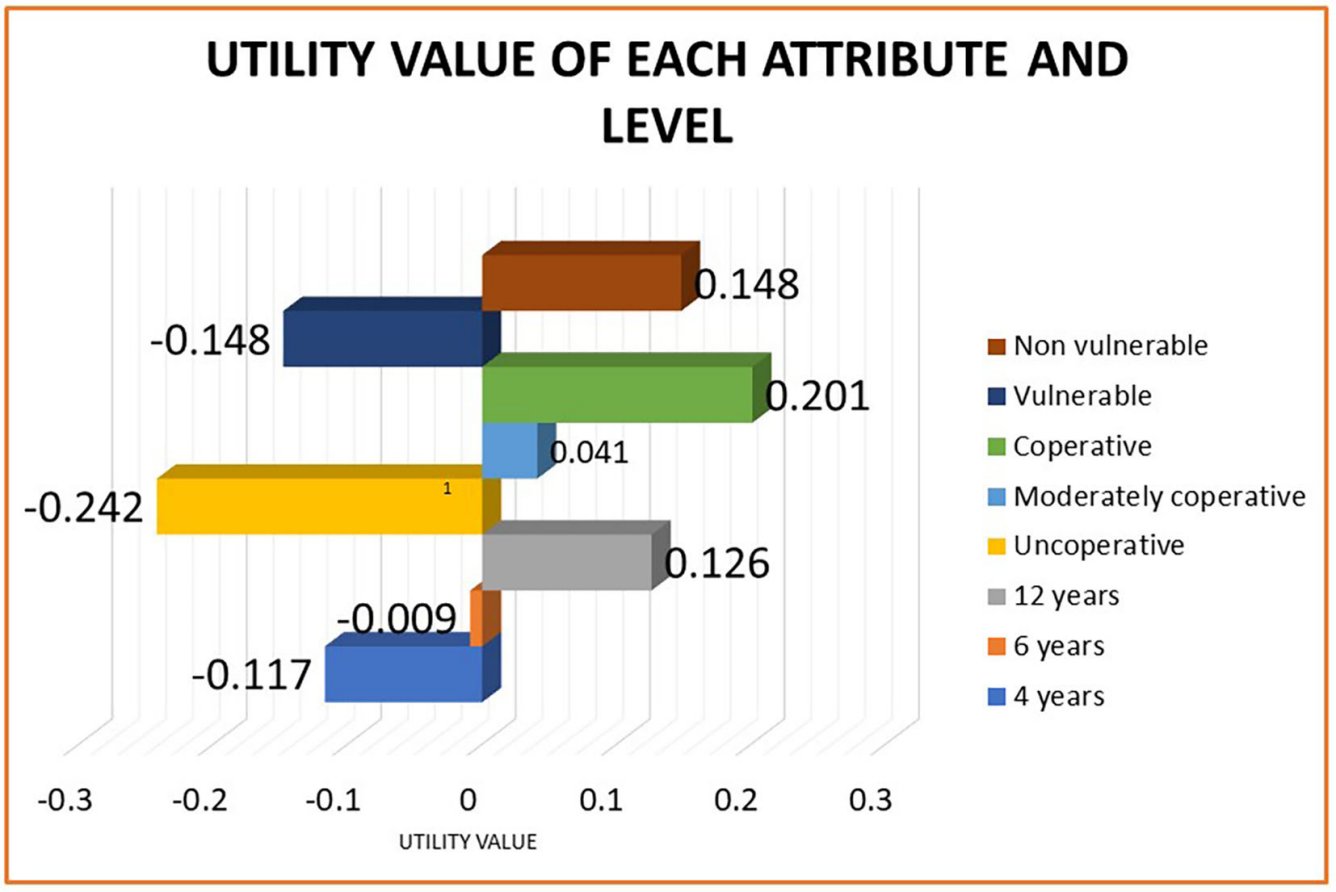
Bar graph depicting the utility value for each attribute levels.

The utility value for each attribute and level was computed. For the attribute cooperation, vulnerability status and age, levels uncooperative, being vulnerable, and four years had the lowest utility values of-0.242, -0.148, and -0.117, respectively ( Figure 3). As a score of one was given for option very likely and five for option very unlikely, the attribute levels with the least value suggest the ones with a high preference for treatment decision. The total utility for different combinations of attribute levels is calculated using an additive linear regression equation ( Table 1). The scenario being uncooperative, belonging to vulnerable section and four years of age was most preferred for ART (2.292). Being moderately cooperative, non-vulnerable, and 12 years old was the least preferred scenario (3.114).

**Table 1.** Preference (utility) scores for the nine case scenarios presented in the conjoint analysis questionnaire.

Scenario	Age	Cooperation level	Vulnerability status	Utility score	Ranking
**Scenario 1**	4	Cooperative	Non-vulnerable	3.031	8
**Scenario 2**	6	Moderately Cooperative	Vulnerable	2.683	4
**Scenario 3**	12	Cooperative	Vulnerable	2.978	7
**Scenario 4**	4	Uncooperative	Vulnerable	2.292	1
**Scenario 5**	12	Uncooperative	Vulnerable	2.535	2
**Scenario 6**	6	Cooperative	Vulnerable	2.843	6
**Scenario 7**	12	Moderately Cooperative	Non-vulnerable	3.114	9
**Scenario 8**	6	Uncooperative	Non-vulnerable	2.696	5
**Scenario 9**	4	Moderately Cooperative	Vulnerable	2.575	3

## Discussion

To our knowledge, this is the first study in India to use a clinical scenario-based and rigorous statistical approach to measure pedodontists’ perception (relative utilities) for decision making for ART. We found that pedodontists value behavior and vulnerability status in decision-making when compared to the age of the child. An uncooperative child belonging to the vulnerable population and being young was the most preferred scenario in clinical decision-making for ART by Pedodontists. The depth of the lesion and individual caries risk were the most important factors reported while considering ART as a definitive treatment.

The use of conjoint design demonstrated the importance of patient factor vulnerability status in clinical restorative treatment decisions for children, in addition to their age and cooperation level. Using conjoint analysis, the relative importance of different factors, trade-offs pedodontists make between these factors, and utility or importance for combinations of test factors were possible. This is because it enables the measurement of psychological, real, or hidden factors in professional behavior (decision-making). ^24^

In the present study, ART was considered an interim treatment by 62% pedodontists for the presented scenarios, similar finding was observed in a previous study where 82% American pedodontists stated the same. ^10^ The views are opposing to that of the current literature wherein ART is viewed globally as a definitive treatment, particularly in certain populations that do not have access to more traditional restorative treatment. ^10^ The World Health Organization and International Dental Federation recognize ART as part of the basic package of oral care for all communities around the world. ^25^

A pre-invitation mail and three attempts were made to contact all the registered pedodontists (1200) through online electronic mail to improve response rates, as stated by Dillman. ^23^ We could achieve a response rate of 35.9%. With a reported average response rate of 24.8% for email surveys ^26^ we achieved a better response. Similar response rates were found in other published surveys by Janakiram C (30.9%) and Kaul R (38.3%). ^27, 28^ Despite having active email accounts, online surveys were not acceptable to many. ^27^ In our study about 432 pedodontists participated. Al-Omari B et al ^20^ recommended a sample size of 300 for conjoint study. Therefore, the study sample size was larger than the recommended sample size for the conjoint analysis.

Controlled clinical factors independently drive the decision to administer ART. It enabled assessment of the pedodontists’ perception and their relative importance on the other three non-clinical factors in clinical decision-making. The clinical presentation was fixed in accordance with recent evidence showing that ART single-surface restorations using high-viscosity GIC had a high survival rate in both primary and permanent teeth, comparable to that of traditional amalgam restorations. ^29^ Moreover, as ART restorations are both minimally invasive and caries-protective when compared to other traditional restorative methods, it can be considered as a treatment of choice for single-surface caries lesions. ^30^

In the present study, ART was more likely to be preferred by young children who were uncooperative and vulnerable. These findings are consistent with the endorsement of the World Health Organization and the International Association for Dental Research. According to the WHO, ART may be used to restore and prevent caries in young patients, uncooperative patients, or patients with special health care needs ^31^ and also where traditional cavity preparation and/or placement of traditional dental restorations are not feasible. ^31^ As stated by Jo E Frenken, ART is the best treatment for very young children with behavioral problems. ^32^ The results are in accordance with the study by Kateeb et al., where the pediatric dentist preferred ART for uncooperative children. ^10^ Interestingly, in the present study, young age had less utility value and the last preferred factor among the three non-clinical factors. In contrast, young age was the second most preferred factor for ART treatment in a study by Kateeb et al. ^10^

The vulnerability status factor was considered to identify the relative importance of pedodontists in clinical decision-making. The results showed that the vulnerability status of the child was the second most important factor stated by study participants. Even though Atraumatic Restorative Treatment (ART) is considered an economical and effective method for preventing and controlling carious lesion development in vulnerable populations. ^18, 33^ The recent evidence stated higher survival rates comparable to traditional restorations and being safe to use. ^33^ ART can be considered to be a cornerstone of minimal intervention caries management in combination with prevention and minimal invasion. ^30^ Therefore, thorough and careful performance of all the technical steps can lead to successful long-lasting restorations. Further studies on atraumatic restorative treatment are needed to validate its effectiveness and acceptability.

A study by Molina et al. showed a higher one-year survival rate for all ART restorations compared to conventional restorations in adults with intellectual disability. ^34^ The strengths of the study were the joint design, which allowed an unbiased precise preference estimate, thereby reducing confounding bias. Anonymous mailed questionnaires reduced interviewer bias and social desirability bias. The survey had a few scenarios and questions, thereby reducing response fatigue. A subset of potential patient scenarios was randomly selected and presented to participants using an orthogonal fractional factorial design. The software algorithm was programmed to extrapolate the total utility values for all possible patient scenarios for each participant. It generates a random sequence of scenarios, avoiding learning and primacy biases.

The hypothetical nature of questions, time consumption, and reported behavior may not be the actual behavior, which were the limitations of the study. Further studies are required to confirm the hypotheses derived from this study. Dental professionals should be encouraged to adopt new, less invasive, and simple restorative techniques, such as ART. Pedodontists can be sensitized to the effectiveness of ART, irrespective of children’s cooperation, vulnerability status, and age. This can be emphasized by incorporation into the early stages of clinical training through CDE and CME programs.

## Conclusion

The most preferred scenario by pedodontists to consider ART as the treatment of choice was an uncooperative child belonging to a vulnerable section and being very young.

## Ethics and consent

The study protocol was approved by the Institutional Review Board of Bapuji Dental College and Hospital, under approval number BDC/383/2017-18 dated 24/11/2017. The Committee on Research Ethics adhered to international guidelines for human subject protection, including the Declaration of Helsinki, The Belmont Report, and CIOMS guidelines. All participants provided voluntary written informed consent using an electronic consent system before accessing the online survey. The consent form clearly outlined the survey’s purpose, the estimated time required to complete the questionnaire, and used simple, understandable language. Participants were informed that they could withdraw from the survey at any time, and that their personal information would be kept confidential.

## Data Availability

Figshare – Understanding decision making for use of Atraumatic Restorative Approach based on non-clinical factors by Indian Pedodontists - A conjoint analysis, https://doi.org/10.6084/m9.figshare.27188796. ^35^ This project contains following underlying data:
• Conjoint study-data.xlsx Data are available under the terms of the Creative Commons Zero “No rights reserved” data waiver (CC BY 4.0 Public domain dedication). ^35^ Figshare - Understanding decision making for the use of atraumatic restorative approach based on non-clinical factors by Indian pedodontists - a conjoint analysis (Questionnaire), https://doi.org/10.6084/m9.figshare.27283890. ^36^ This project contains following extended data
• Survey questionnaire, participant information and consent form.docx Data are available under the terms of the Creative Commons Zero “No rights reserved” data waiver (CC BY 4.0 Public domain dedication).
