# Supplementary material for: Understanding decision making for the use of atraumatic restorative approach based on non-clinical factors by Indian pedodontists - a conjoint analysis
Source: F1000Res. 2024 Nov 21;13:1401. [Version 1] doi: 10.12688/f1000research.157561.1 (PMC11615434; doi:10.12688/f1000research.157561.1)
Supplement: Peer review history

# Reviewer report: Reviewer response for version 1

**DOI:** 10.5256/f1000research.173021.r343457

**Published:** 2024-12-03

**Authors:** Dr Praveen Gangadharappa

**Affiliations:** 1 Jazan University, Jazan, Saudi Arabia

**Title and Abstract:** The title and abstract effectively summarize the key aspects of the study, giving a clear overview of its scope and objectives. **Introduction:** The introduction presents a well-structured and logical framework, supported by relevant citations. It successfully establishes the background and context for the study, highlighting the need for the research in the field.

**Need for the Study:** The rationale for the study is strongly justified, with a solid scientific background that highlights its significance. The research addresses an important gap in the existing literature, underlining the contribution it makes to the field. • **Methodology:** The methodology is clearly described and easily replicable. The research design, sampling methods, and procedures are well-detailed. The statistical analysis is appropriate and robust, ensuring that the findings are valid and reliable.

**Tables and Figures**: The tables and figures are relevant and well-aligned with the objectives of the study. They help in presenting the data clearly and support the interpretation of the results.

**Discussion:** The discussion is meaningful and provides a thorough interpretation of the results. The comparison with previous studies is appropriate and helps to contextualize the findings within the broader scientific landscape. 

**Conclusion:** The conclusion is consistent with the study's findings. It clearly summarizes the key outcomes and provides insights that are logically derived from the research.

Is the work clearly and accurately presented and does it cite the current literature?

Yes

If applicable, is the statistical analysis and its interpretation appropriate?

I cannot comment. A qualified statistician is required.

Are all the source data underlying the results available to ensure full reproducibility?

Yes

Is the study design appropriate and is the work technically sound?

Yes

Are the conclusions drawn adequately supported by the results?

Yes

Are sufficient details of methods and analysis provided to allow replication by others?

Yes

Reviewer Expertise:

IMPLANTS, IMPRESSIONS, COMPLETE DENTURE PROSTHODONTICS,

I confirm that I have read this submission and believe that I have an appropriate level of expertise to confirm that it is of an acceptable scientific standard.

# Reviewer report: Reviewer response for version 1

**DOI:** 10.5256/f1000research.173021.r343455

**Published:** 2024-12-02

**Authors:** Dr Suresh KV

**Affiliations:** 1 SEGi University Kota Damansara, Petaling Jaya, Selangor, Malaysia

Thank you for this opportunity  

 The manuscript is well-structured and adheres to the STROBE guidelines, ensuring clarity and transparency in reporting. Both the title and abstract are informative, providing a concise summary of the study’s aims, methods, and key findings. The introduction effectively highlights the need for the study, clearly stating the research aim and objectives. The background provided justifies the significance of the study in the current scientific context. The authors have thoroughly explained the validation process for the data collection instrument, ensuring reliability and credibility. The study design is well-described, and the sample size has been scientifically estimated using an appropriate formula. The depth of detail in the method of data collection facilitates replication, making the research transparent and reproducible. The consent process is described in detail, ensuring adherence to ethical standards in research. The statistical methods used for analysis are clearly specified and are appropriate for the type of data obtained. This ensures the robustness of the results and provides confidence in the conclusions drawn. The results are clearly presented in well-organized tables, with comprehensive information about the characteristics of the study participants. This allows for a detailed understanding of the study's sample and its generalizability. The discussion provides a concise summary of the key results and interprets them within the context of previous literature. The authors have appropriately cited studies with similar findings, providing a balanced and informed discussion. The manuscript includes useful recommendations for conducting similar research in the future, based on the study's findings and limitations. References are following the journal’s guidelines.

Is the work clearly and accurately presented and does it cite the current literature?

Yes

If applicable, is the statistical analysis and its interpretation appropriate?

Yes

Are all the source data underlying the results available to ensure full reproducibility?

Yes

Is the study design appropriate and is the work technically sound?

Yes

Are the conclusions drawn adequately supported by the results?

Yes

Are sufficient details of methods and analysis provided to allow replication by others?

Yes

Reviewer Expertise:

TMDs, Orofacial pain, CBCT

I confirm that I have read this submission and believe that I have an appropriate level of expertise to confirm that it is of an acceptable scientific standard.
